# Caught between confidentiality and compulsion: the global ethics-law dilemma in psychotherapy

**DOI:** 10.3389/fpsyg.2025.1665158

**Published:** 2026-01-12

**Authors:** Simi John, Biju P. Mani, Binny Jose

**Affiliations:** 1 Department of Law, Marian College Kuttikkanam Autonomous, Kuttikkanam, India; 2 Department of Health and Wellness, Marian College Kuttikkanam Autonomous, Kuttikkanam, India

**Keywords:** confidentiality, mandatory reporting, ethical-legal tension, psychotherapy, comparative analysis

## 1 Introduction

The core of psychotherapy is confidentiality. Clients only share their traumatic experiences when they know their concerns will be protected. The ethical underpinnings of confidentiality are increasingly being challenged by legislation requiring psychotherapists to disclose client information—typically child abuse, suicide intent or violent intentions. Such legislative interventions are typically driven by a compelling social imperative to prevent ongoing harm, address severe power asymmetries, and respond to historical failures of institutions to protect children and other at-risk individuals from abuse, exploitation, or life-threatening neglect. Although the intent of this legislation was to protect vulnerable populations, these statutory requirements present a problem for psychotherapists who must choose between maintaining client confidentiality and fulfilling legal responsibilities which may undermine that confidentiality.

This dilemma exists globally. Different jurisdictions have developed different methods of balancing the rights of clients with the need for mandated reporting. In many countries including India's Protection of Children from Sexual Offenses Act (Pocso, 2012) Australia's Care and Protection of Children Act and U.S. State level child protective service statues require broad reporting obligations that take precedence over the therapist-client privilege (Tufford, 2019). Other jurisdictions, such as Canada and several Australian jurisdictions provide for discretionary or conditional privilege (Walsh and Mathews, 2014; Walters, 1995).

This paper provides a comparative legal-ethical analysis of India, the United States, the United Kingdom, Australia and Canada—jurisdictions that represent varying legal traditions and levels of discretion. Through an examination of the laws governing each jurisdiction, the ethical guidelines of each jurisdiction and case precedent in each jurisdiction, the authors identify areas of conflict and potential for harmonization.

The ethical discussion is based on the principles of autonomy, beneficence and non-maleficence (Cram and Dobson, 1993; Weinstock and Weinstock, 1989) and places these principles within broader discussions regarding deontological vs. consequentialist reasoning. Mandatory reporting laws can limit the number of services available to patients (Gilbert, 1986; Wolkowitz, 2024).

This opinion article synthesizes legal, ethical, and clinical perspectives to propose reforms that balance confidentiality with public protection. The next section describes the method used to compare the legal, ethical and clinical aspects of the jurisdictions discussed in this paper.

## 2 Methodology

Using a comparative legal-ethical framework, this study examines the way that India, the USA, UK, Canada and Australia manage the relationship between therapeutic confidentiality and the requirement for mandated reporting. These jurisdictions were chosen in part because they reflect different legal paradigms (statutory, common law and hybrid systems) and cultural attitudes toward both the autonomy of professionals and the protection of the public. Collectively, these five jurisdictions offer a range of approaches that illustrate both mandatory and discretionary components of mental health law.

### 2.1 Selection criteria

The studies drew primarily on three types of information resources:

(a) Statutes and court decisions relating to the confidentiality of therapists and clients, and the obligation to report;
(b) Codes of ethics of the professional bodies of psychologists and psychiatrists in each country; and,
(c) Peer reviewed academic research in the areas of law, ethics and mental health practice.

Only jurisdictions with (a) codified statutes requiring therapists to make reports of client abuse or neglect when required by law, (b) codes of ethics that outline the duty of confidentiality, and (c) evidence of debate among the professions regarding issues of ethical and legal compliance with respect to the obligations to protect the public from harm were considered for inclusion in the study.

### 2.2 Analytical approach

Therapeutic and ethical materials were analyzed using textual and thematic analysis to identify key concepts including privilege, discretion and harm prevention. Subsequently, the texts were compared based on their jurisprudence relative to a continuum of reporting from rigid to discretionary reporting frameworks. In addition, the study applied ethical theories including autonomy, beneficence and non-maleficence to the public health implications associated with the reporting requirements.

### 2.3 Epistemic scope

As an opinion piece, this study did not involve the collection of new empirical data. Rather, it provided an interpretative and normative critique developed through an integration of statutory and regulatory materials, ethical guidelines and peer-reviewed literature. The purpose was to identify conceptual and policy tensions rather than empirically quantify the prevalence of these tensions.

## 3 The ethical foundation of confidentiality in therapy

Privacy is a basis for ethical practice in both psychology and psychiatry. Clients will only share their most intimate thoughts and feelings with a therapist if they can be confident that the therapist will protect their confidences. Confidentiality is therefore a requirement for building trust, and for allowing the therapeutic relationship to develop (Cram and Dobson, 1993).

Confidentiality is recognized as an important element of the major ethical frameworks for counseling and psychotherapy in the UK (British Association for Counseling and Psychotherapy's Ethical Framework), and in the USA (American Psychological Association's Ethical Principles). Both recognize confidentiality as a key component of respecting clients' autonomy, and maintaining clients' safety.

There are three key ethical bases upon which confidentiality rests: autonomy, beneficence, and non-maleficence (Weinstock and Weinstock, 1989). Autonomy respects the client's right to decide who has access to their personal information. Beneficence and non-maleficence (the duty to act in ways that promote the wellbeing of others and to avoid causing them harm) mean that confidentiality is used to promote the well-being of the client and to avoid harming them. Therefore, confidentiality is not just a matter of procedure: it gives the client back their sense of agency and dignity.

Deontologically speaking, confidentiality is a reflection of a duty to respect persons. Breach of trust represents a violation of a person's moral worth from a Kantian perspective. Consequentialism would suggest that there may be circumstances under which it is permissible to breach confidentiality to prevent harm to others, or to promote the greater good. However, this presents therapists with a difficult task: to balance their obligations to the client and to society. As noted by Beauchamp and Childress (2019), ethical decision making is about finding a way to balance conflicting principles, and not simply about being faithful to one principle at the expense of another.

Wherever mental health stigma continues to exist, confidentiality is often the door through which people enter into care. Privacy is identified by the World Health Organization as essential for equitable access to care for all individuals (Mental Health Action Plan, 2013). Rigid laws that restrict a therapist's ability to exercise discretion in relation to confidentiality, can result in fewer people seeking help, and a breakdown in the therapeutic relationship.

The vast majority of ethical codes allow for some limitations to confidentiality, such as imminent risk of serious harm to self or others, but the extent of the limitations will always rest on the professional judgment of the therapist, and not solely on the mandate of law. The conflict between discretion and compulsion, discussed further below, arises when ethical decision making is replaced by legal coercion.

## 4 Legal obligations to disclose: a comparative overview

While the ethical foundation of confidentiality is widely shared, legal cultures differ sharply in balancing privacy against public-safety obligations. Some jurisdictions impose rigid, non-discretionary duties to report; others allow professional judgment or privilege. The Table 1 below summarizes how five representative systems regulate this tension.

**Table 1:** Summary of five representative systems tension.

**Jurisdiction**	**Key law(s)**	**Scope of mandatory reporting**	**Discretion / exemptions**	**Recognition of privilege**	**Notes / Recent developments**
India	*Protection of Children from Sexual Offenses Act* (Pocso, 2012); Mental Health act (2017)	“Any person,” including therapists, must report suspected child sexual abuse (§19–21 POCSO)	None; failure to report is punishable	No psychotherapist–client privilege	Supreme Court in *X v. Govt of NCT Delhi* (2022) permitted anonymized reporting but broader guidance absent
United States	State child-abuse laws; Jaffee v. Redmond (1996); Taraso (1976)	Therapists must report child abuse and imminent threats	Limited professional discretion; varies by state	Federally recognized in *Jaffee v. Redmond*	“Duty to warn” doctrine illustrates balanced breach under imminent-harm standard
United Kingdom	United Kingdom (1989); United Kingdom (1998); United Kingdom (2018)	Safeguarding duties in regulated services; statutory reforms pending	Historically discretionary, moving toward limited mandates	Confidentiality protected under HRA 1998 and DPA 2018	Post-IICSA reforms may formalize mandatory-reporting duties
Australia	State child-protection statutes (e.g., Northern Territory Government, 2007); *Mental Health Act [VIC]*	Designated professionals must report child abuse / neglect	Some state discretion (e.g., Victoria allows contextual judgment)	Partial; inconsistent among states	Royal Commission reforms removed religious confession privilege in several states
Canada	Provincial child-protection acts; R v. Mills (1999)	Duty to report ongoing harm or risk to minors	Conditional discretion via professional judgment	Conditional privilege recognized by courts	Emphasis on informed consent and transparency during intake (Walters, 1995)

### 4.1 Comparative insights

- Rigid frameworks (e.g., India) criminalize non-reporting and eliminate discretion.
- Hybrid systems (U.S., Australia) balance duties with judgment through case law such as Tarasoff.
- Discretion-based systems (Canada, U.K.) embed confidentiality within human-rights or common-law privilege, though reforms are narrowing this space.

These variations reveal a continuum from compulsion to discretion. Where the law dominates, confidentiality becomes fragile; where judgment is preserved, trust endures. The next section illustrates these contrasts through three case studies.

## 5 Case studies of ethical-legal conflict

The following cases illustrate how legal mandates and ethical discretion collide in clinical practice. Drawn from statutes, judicial precedents, and professional scenarios within the five jurisdictions, they demonstrate the human and systemic consequences of mandatory reporting regimes.

### 5.1 Case 1: India—confidentiality vs. statutory duty

The Delhi psychotherapist has learned that one of her clients at 14 years old is currently being sexually violated, however she has begged to be kept confidential. Non-reporting under India's Protection of Children from Sexual Offenses (POCSO) Act 2012 is punishable (Section 21), which leaves the therapist with two options: breach the client's trust or face legal consequences (Batra, 2024; Jagadeesh et al., 2017). The therapist ultimately complies with the law, filing a report that triggers police investigation but ends therapy abruptly. Research also suggests the lack of flexibility within the law discourages adolescents from going to sexual health and counseling services; according to the Center for Health and Social Justice, there has been a reduction in access to contraception and mental health care particularly in rural settings (Wolkowitz, 2024). Therefore, the protection provided by the law may actually undermine the laws' intended purpose.

### 5.2 Case 2: United Kingdom—Ethical discretion

The case of a 23-year-old in London who expresses thoughts of suicide to a therapist is treated as such because although there was no immediate risk of harm, he continued treatment without disclosing the patient's expressed intent to commit suicide to others—consistent with U.K. law and ethics (Wintemute et al., 2018). The British Association for Counseling and Psychotherapy permits the disclosure of this information only when it is believed that an individual may be at “immediate and serious danger.” Research has shown that early or premature reporting can lead to loss of trust and less likely to seek out help and support from mental health professionals (APA, 2017; Weinstock and Weinstock, 1989). In this case, the decision made by the therapist was both ethically correct and legally permissible while maintaining both the safety of the client and confidentiality.

### 5.3 Case 3: Cross-border telehealth dilemmas

The online clinician is now faced with a conflict in their obligations as a licensed clinician because of differing laws and mandates in both states, and by virtue of the fact that the client is located outside the country from which they are licensed. This cross-country jurisdictional conflict is a relatively recent phenomenon for many licensed therapists who are practicing globally via teletherapy. Historically, there has been a gap between the development of legal frameworks regarding clinical practice and the actual use of teletherapy in clinical practice (Barnett and Kolmes, 2016; Lee and Zambelli, 1985; Stoll et al., 2020). The World Health Organization (WHO) and the International Association for Counseling (IAC) have called for the establishment of international guidelines and standards regarding the reporting of sexual abuse/assault in electronic counseling (digital therapy; Hansen and Bach, 2023).

In all three examples above, the imposition of strict mandates results in the suppression of help-seeking behavior among those most likely to be helped, while the exercise of measured clinical discretion promotes open therapeutic relationships. In addition to the negative impact on the therapeutic relationship, when clinicians become enforcers of the law, rather than caregivers of their clients, trust is eroded in the therapeutic process. The subsequent section will explore the broader public health implications of this shift.

## 6 Psychological and public health consequences

Reporting requirements are implemented as a means to protect vulnerable individuals however the mandatory reporting requirement creates unintended psychological and structural ramifications for the individuals affected by it. Mandatory reporting has been shown across various countries to create an environment of apprehension toward receiving assistance and further, damage trust between therapist and client, particularly with adolescent clients and those who have experienced abuse.

### 6.1 Impact on help-seeking and trust

The impact of mandatory reporting requirements is evident in India where the Protection of Children from Sexual Offenses Act (Pocso, 2012) mandates all adults including healthcare providers to report child sexual abuse. As a result, adolescent clients do not disclose their need for sexual health and/or counseling services to avoid the possibility of being reported to law enforcement (Ramanathan et al., 2020; Wolkowitz, 2024). A similar barrier exists in Australia where fear of civil liability prevents many youth and families from disclosing sensitive information (Mah, 2014; Sawrikar and Katz, 2017). Studies conducted in the United States also demonstrate that knowledge of the obligation to report diminishes the likelihood of disclosure regarding suicidal ideation and abuse and limits opportunities for early intervention (Høyen et al., 2021; Rodriguez et al., 2024). Therefore, while laws may be enacted to increase the protection of children, they ultimately limit the willingness of youth to seek help.

### 6.2 Psychological burden on professionals

Clinicians report increased levels of anxiety, moral distress, and concerns about the potential for litigation due to the lack of professional discretion when mandated to report suspected child maltreatment (Letson and Crichton, 2023; Tufford, 2019). The process of ethical decision-making is replaced with legal compliance that results in defensive practice rather than therapeutic practice. The use of surveys conducted in Australia and Canada found that professionals were likely to over-report due to uncertainty regarding the threshold for reporting (Walsh and Mathews, 2014), which places undue burdens on investigative agencies and diverts resources away from higher risk situations.

### 6.3 Systemic and disparities

The impact of mandatory reporting is disproportionate to low-income, Indigenous and immigrant communities that historically experience distrust in institutions (Dettlaff, 2025; McTavish et al., 2019). As a result, there is a greater tendency for these groups to disengage from services when confidentiality appears to be at risk and this contributes to widening disparities in the availability of mental health services (Hui et al., 2021; Humphreys et al., 2025; Lehrer et al., 2007). Additionally, research conducted in Canada and the United States demonstrates that reporting systems contain implicit biases resulting in a higher number of investigations into the lives of marginalized families without a corresponding increase in documented instances of harm (Greenwald et al., 2022; Moscou et al., 2023).

Collectively, these studies demonstrate a paradox of public health policy: laws enacted to protect individuals can inadvertently interfere with providing quality care. To effectively address this issue, reforms must balance the need to maintain safety with the need to maintain trust such that clinicians are able to make ethically informed decisions in a supportable legal framework.

## 7 Toward a harmonized and ethical legal framework

Mandatory reporting protects those who are vulnerable, but it also restricts the use of professional judgment, and access to care when it is applied too strictly. To reform this area, the emphasis should be placed on discretionary practices that allow health professionals to act in an ethical manner without fear of a legal response.

- Tiered reporting models: a tiered reporting model provides professionals with the opportunity to report their concerns in a step-wise fashion, first through internal multidisciplinary review, then through external reporting. For example, a clinic may require that all reports be first reviewed by an ethics or safeguarding committee to ensure compliance with the legal criteria, providing a level of accountability while preserving the therapeutic relationship between the client and the clinician.
- Structured professional discretion: structured discretion has been implemented in some countries, most notably in Canada and Victoria (Australia). It acknowledges the ethical expertise of clinicians, while at the same time, maintains a high degree of oversight. Clinicians may exercise short term deferment of reporting when the clinician believes that the reporting would endanger the client's safety or impede the progress of therapy. All documentation of this decision-making process and subsequent supervisory review will provide for transparency in the decision-making process, and protect against retaliatory action being taken against the clinician.
- Education and public awareness: educational programs for clinicians should include case studies, and peer consultation to assist them in navigating the gray area between the legal and ethical implications of their actions. In addition, public education campaigns may help to clarify the difference between using discretion, and neglect; thereby helping to reinforce decisions that align with human rights.
- Global coordination for digital practice: the increasing trend toward teletherapy across national borders requires international bodies such as the World Health Organization (WHO), and the International Association for Counseling (IAC) to develop cross-national guidelines regarding confidentiality and reporting, and to provide protection for both practitioners and clients.

## 8 Conclusion

Mandatory reporting safeguards the vulnerable but can erode therapeutic trust when it removes clinicians' capacity for ethical discretion. The issue lies not in the act of reporting, but in a responsible manner in which it is done; therefore, we require laws that account for the complexity of ethics and provide a rationale for a decision, as opposed to simply a formula.

In summary, the confidentiality-reporting dilemma has many of the same questions society has about trust, responsibility, and individual autonomy. As such, developing maturity in ethics requires balancing our obligation to protect others with the obligation to allow individuals to be heard. We have the potential to create a legal framework that allows for accountability in a legal context while providing ethical guidelines, and safeguards the rights of individuals in a way that continues to permit therapists to perform their healing roles, not as agents of law.
